# Silent spreaders: multidrug-resistant and hypermucoviscous *Klebsiella* from animals threaten one health

**DOI:** 10.1007/s42770-026-02023-6

**Published:** 2026-08-07

**Authors:** Michelle de Souza Moura Gonçalves, Cássia C. da Motta, Dayanne A. De Melo, Thérèsse C. N. Holmström, Miliane M. S. De Souza

**Affiliations:** 1 Department of Veterinary Microbiology and Immunology, Veterinary Institute, Rio de Janeiro Federal Rural University, UFRRJ BR465 km 07 – Seropédica, Rio de Janeiro, 23890-000 Brazil; 2 Tecsa Laboratory, Rua Mena Barreto, 37 - Botafogo, Rio de Janeiro, 22271-100 Brazil; 3 Medicine Institute, University of Vassouras, Av. Expedicionário Osvaldo de Almeida Ramos, 280 - Centro, Vassouras, Zipcode, 27700-000 Brazil

**Keywords:** *bla*TEM, *bla*CTX, *bla*SHV, *bla*KPC, *bla*NDM, *bla*OXA-48

## Abstract

This study investigated the identification, antimicrobial resistance patterns, and phenotypic and genotypic traits of 30 Klebsiella isolates recovered from diverse animal species and infection sites. MALDI-TOF MS demonstrated higher accuracy in species-level identification than conventional phenotypic approaches, revealing a predominance of Klebsiella pneumoniae (80%), followed *by K. variicola*,* K. aerogenes*,* and K. oxytoca*. Multidrug resistance was detected in 46.6% of isolates, while 43.3% were extended-spectrum β-lactamase (ESBL) producers carrying genes such as blaTEM, blaCTX, and blaSHV. One carbapenemase-producing isolate was confirmed, and carbapenemase-encoding genes (blaOXA-48, blaNDM, blaKPC) were identified in additional strains. Notably, hypermucoviscosity was observed in four isolates, including the first report of a hypermucoviscous *K. variicola* from a dog. These findings highlight the presence of antimicrobial-resistant *Klebsiella* isolates in animal sources and support the need for integrated One Health surveillance.

## Introduction

Members of the order Enterobacterales—notably the genus *Klebsiella*—are frequent etiologic agents of veterinary infections, including urinary and respiratory tract disease, septicemia, and opportunistic nosocomial infections. Therapeutic management commonly relies on advanced β-lactams (third- and fourth-generation cephalosporins), β-lactam/β-lactamase inhibitor combinations, fluoroquinolones, aminoglycosides, and, in refractory cases, carbapenems.

Over recent decades, the global rise of antimicrobial resistance (AMR) has critically undermined these options. In particular, extended-spectrum β-lactamase (ESBL) producers and carbapenemase-producing Enterobacteriaceae have been prioritized by the World Health Organization due to their clinical impact and capacity for rapid dissemination. The selection and spread of resistance determinants are driven by antimicrobial misuse across human, animal, and environmental settings, reinforcing the need for integrated surveillance strategies under a One Health framework.

Within this context, multidrug-resistant (MDR) *Klebsiella* spp. have emerged across humans, companion animals, livestock, and wildlife, raising concerns regarding interspecies transmission and environmental persistence. MDR was defined as resistance to at least one agent in three or more antimicrobial classes [13]. Accurate species identification is essential for epidemiological investigation and infection control, however, conventional biochemical methods may misclassify closely related taxa such as *K. pneumoniae* vs. *K. variicola*. Proteomic identification using MALDI-TOF MS, combined with molecular approaches, has significantly improved taxonomic resolution and diagnostic accuracy.

A further layer of concern is the hypermucoviscosity phenotype described in some *Klebsiella* isolates, which has been associated with increased capsule production and may contribute to enhanced pathogenic potential. However, it is important to emphasize that hypermucoviscosity alone is not sufficient to define a hypervirulent phenotype, which typically requires the presence of specific virulence-associated genes or genomic confirmation.

This study aimed to characterize *Klebsiella* isolates recovered from clinical samples of domestic and wild animals by: (i) refining species identification using MALDI-TOF MS, (ii) determining phenotypic antimicrobial susceptibility profiles and MDR patterns, (iii) detecting ESBL- and carbapenemase-associated genes, and (iv) assessing the hypermucoviscosity phenotype. We hypothesized that *Klebsiella* isolates from animal sources may harbor clinically relevant antimicrobial resistance determinants and phenotypic traits associated with increased pathogenic potential, although no direct inference of hypervirulence was assumed.

## Methodology

### Sampling

Thirty isolates previously identified as *Klebsiella* spp. at the Veterinary Microbiological Diagnostic Laboratory (IV-UFRRJ) and at a private diagnostic laboratory, between 2017 and 2020, were included in this study. The isolates originated from a variety of domestic and wild animal species and were obtained from different clinical sites, including otic, uterine, choanal, and respiratory swabs, as well as skin, urine, lung tissue, and prostatic fluid (CEUA 9378290622**).** A convenience sampling strategy was employed, including all available *Klebsiella* isolates during the study period, which represents a limitation regarding representativeness.

Identification. Isolates were cultured on MacConkey agar and incubated at 35 °C ± 2 °C for 24 h. Colonies presenting typical morphology (pink, shiny, and in some cases mucoid) were selected. Preliminary identification was performed using Gram staining, 3% potassium hydroxide (KOH) testing, and conventional biochemical methods [11], prior to confirmatory analyses.

### Proteomic identification by MALDI-TOF MS

Species confirmation was performed using MALDI-TOF MS at the Institute of Pharmacology and Molecular Biology (INFAR, EPM/UNIFESP). Isolates were cultured on CLED agar under standardized conditions. Sample preparation followed the formic acid extraction protocol, and spectra were acquired using a Microflex LT mass spectrometer (Bruker Daltonics) operating with a 337 nm nitrogen laser in linear mode. Spectra were collected within the 2,000–20,000 m/z range and analyzed using MALDI Biotyper software (version 2.0, Bruker), with identification based on comparison against the manufacturer’s reference database. Identification scores ranged from 0 to 3, with values ≥ 2.0 considered reliable for genus-level identification and higher scores indicating increased confidence at the species level. Quality control procedures were conducted according to manufacturer recommendations.

### Antimicrobial resistance disk diffusion tests

All isolates underwent phenotypic resistance testing by the disk diffusion method on Mueller-Hinton agar, according to CLSI VET (2022) and BrCAST (2022) protocols. Antimicrobials were selected for evaluation of multidrug resistance (MDR), extended-spectrum beta-lactamase (ESBL) production, and carbapenemase production. MDR: Amoxicillin-clavulanic acid (30 µg) (Penicillins), Aztreonam (30 µg) (Monobactams), Cefepime (30 µg), Cefotaxime (30 µg), Ceftriaxone (30 µg), Ceftazidime (30 µg) (Cephalosporins), Eenrofloxacin (5 µg), Marbofloxacin (5 µg) (Fluoroquinolones), Gentamicin (10 µg) (Aminoglycosides), Sulfamethoxazole-trimethoprim (25 µg) (Sulfonamides), and Tetracycline (30 µg) (Tetracyclines). ESBL: Cefotaxime (30 µg), Ceftriaxone (30 µg), Ceftazidime (30 µg), and Aztreonam (30 µg). Carbapenemase: Meropenem (10 µg).

### ESBL detection

Screening for ESBL production was performed using third-generation cephalosporins and aztreonam. Confirmatory testing was conducted using the combined disk method with clavulanic acid, and interpretation followed CLSI VET [1] criteria.

### Carbapenemase enzyme detection (Carba NP Test)

For the test, isolates were subcultured on 5% sheep blood agar and incubated at 35 °C ± 2 °C for 24 h. Using a calibrated loop (10 µl), a bacterial inoculum was added to a microtube containing 5 M NaCl and vortexed for 1 min, then kept at room temperature for 30 min. A 100 µl solution containing Carba NP + imipenem + phenol red was added to each tube and incubated at 35 °C ± 2 °C for 2 h. Readings were taken at 15 min, 1 h, and 2 h. A positive result was indicated by a yellow/orange color, while a negative result retained the red color [1, 4].

### Hypermucoviscosity phenotype test

Isolates were subcultured on MacConkey Agar and incubated at 35 °C ± 2 °C for 24 h. A sterile pipette tip was touched to the colony to observe the formation of a viscous string. The hypermucoviscosity phenotype was confirmed when the stretched string measured more than 5 mm ([5] adapted).

### Genotypic detection of ESBL and Carbapenemase

For ESBL detection, the genes *bla*TEM [14], *bla*CTX-M [8], and *bla*SHV [17] were investigated. For carbapenemase detection, the genes *bla*KPC, *bla*NDM and *bla*OXA-48 were investigated in multiplex PCR [15] (Table 1).

**Table 1:** Primers used in the characterization of antimicrobial resistance

Gene	Primer	Cicle
*bla*_TEM_ (831 pb)	ATGAGTATTCAACATTTCCGTG TTACCAATGCTTAATCAGTGAG	94 °C for 5 min; 40 **cycles** (94 °C for 1 min, 55 °C for 1 min and 72 °C for 1 min) and final extension for 72 °C for 5 min.
*blaCTX* (862 pb)	AAAAATCACTGCGCCAGTTC CCGTCGGTGACGATTTTAGCC	
*bla*_SHV_ (831 pb)	TTTATCGGCCCTCACTCAAGG TTACCAATGCTTAATCAGTGAG	94 °C for 3 min; 32 **cycles** (94 °C for 30 seg, 56 °C for 30 seg and 72 °C for 1 min) and final extension for 72 °C for 10 min.

### Statistic

Descriptive statistics were used to summarize the data. Proportions were calculated for resistance profiles and phenotypic traits.

## Results and discussion

### Origin of Isolates

Of the 30 *Klebsiella* isolates analyzed, 83.3% (25/30) were originally identified at the Veterinary Microbiological Diagnostic Laboratory of IV-UFRRJ, while 16.6% (5/30) were obtained from a private diagnostic facility. The majority (60%, 18/30) were isolated from dogs, followed by cats (23.3%, 7/30), birds (6.6%, 2/30), rabbit (3.3%, 1/30), cattle (3.3%, 1/30), and crab-eating raccoon (*Procyon cancrivorus*) (3.3%, 1/30). With respect to infection sites, urinary tract samples were the most frequent source (53.3%, 16/30), particularly from dogs (11/16). Other sources included the uterus (13.3%, 4/30), skin (10%, 3/30), respiratory tract (10%, 3/30), ears (10%, 3/30), prostate (3.3%, 1/30), and rectal mucosa (3.3%, 1/30).

These findings corroborate earlier reports highlighting *Klebsiella* as a common etiological agent of urinary tract infections in dogs [12, 18]. The predominance of urinary isolates emphasizes the role of *Klebsiella* in canine urogenital infections, while sporadic cases in wildlife underscore the potential for broader ecological dissemination.

### Species Identification

Phenotypic biochemical methods frequently misclassified isolates, with discrepancies observed in 8/30 cases. In contrast, MALDI-TOF MS provided more accurate identification, confirming *K. pneumoniae* as the predominant species (80%, 24/30), followed by *K. variicola* (13.3%, 4/30), *K. aerogenes* (3.3%, 1/30), and *K. oxytoca* (3.3%, 1/30). All isolates yielded high-confidence genus-level scores (> 2,000), with nearly half (46.6%) achieving highly probable species-level identification.

The misidentification of *K. variicola* as *K. pneumoniae*, documented in previous studies [6, 16], was also observed here. This reinforces the superiority of proteomic methods, which remain crucial for distinguishing closely related *Klebsiella* species in veterinary diagnostics. All isolates scored above 2,000, indicating high reliability for genus-level identification of *Klebsiella*, according to established criteria (Table 2).

**Table 2:** Relationship of isolates with the site of infection, animal species involved, phenotypic identification, proteomic identification and Score

Strain	Infeccion site	Animal	Phenotipic	Proteomic	Score
01	Ear	Dog	*Klebsiella pneumoniae*	*Klebsiella pneumoniae*	2.103 (++)
02	Urinary tract	Dog	*Klebsiella pneumoniae*	*Klebsiella pneumoniae*	2.388 (+++)
03	Urinary tract	Dog	*Klebsiella pneumoniae*	*Klebsiella pneumoniae*	2.185 (++)
04	Urinary tract	Dog	*Klebsiella pneumoniae*	*Klebsiella variicola*	2.243 (++)
05	Skin	Cat	*Klebsiella aerogenes*	*Klebsiella variicola*	2.365 (+++)
06	Urinary tract	Dog	*Klebsiella pneumoniae*	*Klebsiella pneumoniae*	2.206 (++)
07	Urinary tract	Dog	*Klebsiella aerogenes*	*Klebsiella pneumoniae*	2.287 (++)
09	Ear	Dog	*Klebsiella pneumoniae*	*Klebsiella pneumoniae*	2.293 (++)
10	Prostate	Dog	*Klebsiella pneumoniae*	*Klebsiella pneumoniae*	2.405 (+++)
14	Urinary tract	Dog	*Klebsiella pneumoniae*	*Klebsiella pneumoniae*	2.314 (+++)
15	Urinary tract	Dog	*Klebsiella pneumoniae*	*Klebsiella pneumoniae*	2.387 (+++)
16	Urinary tract	Cat	*Klebsiella pneumoniae*	*Klebsiella pneumoniae*	2.145 (++)
17	Respiratory tract	Macaw	*Klebsiella pneumoniae*	*Klebsiella variicola*	2.146 (++)
18	Skin	Cat	*Klebsiella pneumoniae*	*Klebsiella pneumoniae*	2.315 (+++)
19	Urinary tract	Cat	*Klebsiella pneumoniae*	*Klebsiella pneumoniae*	2.240 (++)
20	Urinary tract	Cat	*Klebsiella pneumoniae*	*Klebsiella pneumoniae*	2.020 (++)
22	Uterus	Dog	*Klebsiella pneumoniae*	*Klebsiella pneumoniae*	2.379 (+++)
25	**Respiratory tract**	Rabbit	*Klebsiella pneumoniae*	*Klebsiella pneumoniae*	2.477 (+++)
26	Uterus	Dog	*Klebsiella pneumoniae*	*Klebsiella pneumoniae*	2.387 (+++)
27	Urinary tract	Dog	*Klebsiella pneumoniae*	*Klebsiella pneumoniae*	2.392 (+++)
28	Respiratory tract	Cow	*Klebsiella pneumoniae*	*Klebsiella pneumoniae*	2.297 (++)
29	Uterus	Dog	*Klebsiella pneumoniae*	*Klebsiella pneumoniae*	2.051 (++)
31	Urinary tract	Dog	*Klebsiella pneumoniae*	*Klebsiella pneumoniae*	2.406 (+++)
32	Urinary tract	Cat	*Klebsiella pneumoniae*	*Klebsiella oxytoca*	2.244 (++)
33	Urinary tract	**Dog**	*Klebsiella pneumoniae*	*Klebsiella pneumoniae*	2.424 (+++)
39	Urinary tract	Cat	*Klebsiella pneumoniae*	*Klebsiella pneumoniae*	2.145 (++)
40	Urinary tract	Dog	*Klebsiella oxytoca*	*Klebsiella pneumoniae*	2.265 (++)
41	Uterus	Dog	*Klebsiella pneumoniae*	*Klebsiella variicola*	2.143 (++)
42	Rectum	Crested caracara	*Klebsiella pneumoniae*	*Klebsiella aerogenes*	2.434 (+++)
44	Skin	caracara	*Klebsiella pneumoniae*	*Klebsiella pneumoniae*	2.367 (+++)

Among the 24 *Klebsiella pneumoniae* isolates, infection sites were distributed as follows: urinary tract, 58.3%; uterus, 12.5%; ear, 8.3%; respiratory tract, 8.3%; dermatologic sites, 8.3%; and prostate, 4.1% (Table 3).

**Table 3:** Number of *Klebsiella* spp. isolates according to the site of infection

Klebsiella pneumoniae		Klebsiella variicola	Klebsiella aerogenes	Klebsiella oxytoca
Urinary tract	14	1	0	1
Uterus	3	1	0	0
Skin	2	1	0	0
Respiratory tract	2	1	0	0
Ear	2	0	0	0
Rectum	0	0	1	0
Prostate	1	0	0	0
Total	**24**	**4**	**1**	**1**

### Antimicrobial resistance profiles

Disk diffusion testing revealed that 46.6% (14/30) of isolates were multidrug-resistant (MDR), with resistance to three or more antimicrobial classes. Resistance rates were particularly high against third-generation cephalosporins: cefotaxime (66.6%), ceftazidime (63.3%), and ceftriaxone (53.3%). Notably, one isolate exhibited carbapenem resistance (3.3%).

Screening for extended-spectrum β-lactamase (ESBL) production identified 83.3% (25/30) as potential ESBL producers. Confirmatory testing demonstrated that 43.3% (13/30) met CLSI VET (2022) criteria for ESBL production. Molecular analysis further revealed that 60% (15/25) of screening-positive isolates harbored ESBL-encoding genes: *blaTEM* (66.7%), *blaCTX* (26.7%), and *blaSHV* (6.6%).

These data align with prior studies in domestic animals across Europe, Asia, and South America, which have reported escalating rates of MDR and ESBL-producing *K. pneumoniae* [3, 10, 19]. The rising prevalence observed here reflects a growing threat within veterinary contexts and reinforces concerns over the zoonotic potential of ESBL-producing *Klebsiella*.

### Carbapenemase-producing strains

The adapted Carba NP test confirmed one carbapenemase-producing *Klebsiella pneumoniae* isolate recovered from canine urine, which also exhibited multidrug-resistant (MDR) and extended-spectrum β-lactamase (ESBL) phenotypes. In addition, six isolates were found to carry carbapenemase-encoding genes, including blaOXA-48 (50%), blaNDM (33.3%), and blaKPC (16.7%). Notably, two isolates simultaneously harbored blaCTX and blaOXA-48.

A discrepancy was observed between the genotypic detection of carbapenemase genes and the phenotypic confirmation of carbapenemase production, as only one isolate was confirmed by the Carba NP test. This finding may be explained by several factors, including variable or low-level gene expression, the presence of non-functional or truncated genes, and differences in regulatory mechanisms affecting enzyme production. In addition, phenotypic assays such as the Carba NP test may have limited sensitivity for detecting certain carbapenemases, particularly OXA-48-like enzymes, which are known to confer weak hydrolytic activity and may result in borderline or negative phenotypic results.

It is also important to consider that the mere presence of resistance genes does not necessarily translate into clinically relevant resistance, as gene expression, bacterial fitness costs, and permeability factors (such as porin loss or efflux pump activity) can influence the phenotypic outcome. Therefore, the detection of carbapenemase genes should be interpreted with caution, particularly in the absence of complementary molecular or functional analyses.

Although reports of carbapenem-resistant *Klebsiella* spp. in veterinary medicine remain limited, their detection is of concern given the critical importance of carbapenems as last-resort antimicrobials in human medicine. Similar findings reported in Europe, Asia, and Africa [9, 20] suggest the potential for global dissemination of these resistance determinants. However, due to the lack of additional molecular characterization in the present study, including plasmid analysis or whole genome sequencing, it is not possible to determine the genetic context or transmission dynamics of the detected genes, which represents an important limitation to be addressed in future investigations (Table 4).

**Table 4:** Number of *Klebsiella* spp. isolates that exhibited resistance according to the disk diffusion antibiogram

Antimicrobial	Strains	Percentage
Amoxicilin+ Clavulanic acid	14	46,6%
Aztreonam	19	63,3%
Cefepim	15	50%
Cefotaxim	20	66,6%
Ceftriaxone	16	53,3%
Ceftazidime	19	63,3%
enrofloxacin	13	43,3%
Gentamicin	14	46,6%
Marbofloxacine	9	30%
Meropenem	1	3,3%
Sulfametoxazol-Trimetoprime	11	36,6%
Tetracicline	12	40%

### Hypermucoviscosity phenotype

Four isolates (13.3%) tested positive for the hypermucoviscosity (Hmv) phenotype, including three *Klebsiella pneumoniae* isolates from canine urinary tract infections and one *Klebsiella variicola* isolate recovered from a uterine infection in a dog diagnosed with pyometra. Notably, two of these Hmv-positive isolates also exhibited multidrug-resistant (MDR) and extended-spectrum β-lactamase (ESBL) phenotypes, suggesting a concerning overlap between resistance and phenotypic traits potentially associated with increased virulence.

The identification of a hypermucoviscous *K. variicola* isolate in an animal host represents, to our knowledge, the first report in veterinary medicine. Previous studies describing Hmv-positive K. variicola have been limited to human clinical settings [2, 7], which highlights the potential expansion of this phenotype across hosts and reinforces its relevance within a One Health perspective. However, it is important to emphasize that the hypermucoviscosity phenotype alone is not sufficient to classify isolates as hypervirulent. The absence of molecular characterization targeting virulence-associated genes, such as rmpA, rmpA2, iucA, or iroB, as well as the lack of whole genome sequencing, limits the ability to establish a definitive link between the observed phenotype and hypervirulence.

Furthermore, the coexistence of hypermucoviscosity with MDR and ESBL phenotypes in some isolates may represent a clinically relevant combination, potentially impacting treatment outcomes. Nevertheless, this association should be interpreted with caution, as the genetic basis and expression of both virulence and resistance determinants were not investigated in depth in this study. Additional molecular analyses are required to clarify whether these traits are co-located on mobile genetic elements, such as plasmids, or represent independent characteristics. Overall, these findings underscore the need for further genomic and functional studies to better understand the clinical and epidemiological significance of hypermucoviscous Klebsiella spp. in veterinary contexts.

### One health implications

This study demonstrates the co-occurrence of MDR, ESBL production, carbapenemase-associated genes, and the hypermucoviscosity phenotype in *Klebsiella* isolates from diverse animal species. The findings suggest that companion animals may harbor clinically relevant *Klebsiella* isolates with resistance determinants of veterinary and public health interest. The data highlight the necessity of continuous surveillance, molecular monitoring, and integrated One Health strategies to mitigate the dissemination of these pathogens and safeguard antimicrobial efficacy.

## Conclusion

MALDI-TOF MS outperformed conventional phenotypic methods for species-level identification of veterinary Klebsiella, revealing a predominance of *K. pneumoniae* and the presence of *K. variicola*. Nearly half of the isolates were multidrug-resistant, 43.3% produced ESBLs, and carbapenemase determinants (blaOXA-48, blaNDM, blaKPC) were detected, underscoring the presence of important resistance mechanisms in animal isolates.

Hypermucoviscosity was identified in four isolates, including the first report of hypermucoviscous *K. variicola* from a dog. However, because hypermucoviscosity alone does not confirm hypervirulence, further studies including virulence-associated gene detection and genomic analyses are needed to clarify the clinical significance of these isolates.

Future investigations will build upon these findings by expanding sample collection to a broader, multicenter framework, enabling a more comprehensive assessment of the epidemiological distribution of Klebsiella spp. Additionally, the application of whole-genome sequencing will provide deeper insights into clonal relationships, resistance determinants, and virulence profiles, contributing to a more detailed understanding of the genetic and epidemiological dynamics of these isolates.
